# Neonatal hyperinsulinemic hypoglycemia: case report of kabuki syndrome due to a novel *KMT2D* splicing-site mutation

**DOI:** 10.1186/s13052-020-00902-8

**Published:** 2020-09-18

**Authors:** Ettore Piro, Ingrid Anne Mandy Schierz, Vincenzo Antona, Maria Pia Pappalardo, Mario Giuffrè, Gregorio Serra, Giovanni Corsello

**Affiliations:** 1 Department of Health Promotion, Mother and Child Care, Internal Medicine and Medical Specialties “G. D’Alessandro”, University Hospital “P.Giaccone”, University of Palermo, Piazza delle Cliniche, 2, 90127, Palermo, Italy; 2 Pediatric Radiology Unit, A.R.N.A.S. Ospedali Civico Di Cristina Benfratelli, Piazza N. Leotta, 4, 90127, Palermo, Italy

**Keywords:** Facial dysmorphism, Neonatal hypoglycemia, Hyperinsulinism, Neonatal hypotonia, Nervous system malformation

## Abstract

**Background:**

Persistent neonatal hypoglycemia, owing to the possibility of severe neurodevelopmental consequences, is a leading cause of neonatal care admission. Hyperinsulinemic hypoglycemia is often resistant to dextrose infusion and needs rapid diagnosis and treatment. Several congenital conditions, from single gene defects to genetic syndromes should be considered in the diagnostic approach. Kabuki syndrome type 1 (MIM# 147920) and Kabuki syndrome type 2 (MIM# 300867), can be associated with neonatal hyperinsulinemic hypoglycemia.

**Patient presentation:**

We report a female Italian (Sicilian) child, born preterm at 35 weeks gestation, with persistent hypoglycemia. Peculiar facial dysmorphisms, neonatal hypotonia, and cerebellar vermis hypoplasia raised suspicion of Kabuki syndrome. Hyperinsulinemic hypoglycemia was confirmed with glucagon test and whole-exome sequencing (WES) found a novel heterozygous splicing-site mutation (c.674-1G > A) in *KMT2D* gene. Hyperinsulinemic hypoglycemia was successfully treated with diazoxide. At 3 months corrected age for prematurity, a mild global neurodevelopmental delay, postnatal weight and occipitofrontal circumference growth failure were reported.

**Conclusions:**

Kabuki syndrome should be considered when facing neonatal persistent hypoglycemia. Diazoxide may help to improve hyperinsulinemic hypoglycemia. A multidisciplinary and individualized follow-up should be carried out for early diagnosis and treatment of severe pathological associated conditions.

## Introduction

The Pediatric Endocrine Society suggests a plasma glucose concentration of 50 mg/dL (2.8 mmol/L), corresponding to 45 mg/dL in whole blood, or less as an appropriate threshold to trigger further diagnostic testing in a child less than 48 h old, and 60 mg/dL (3.3 mmol/L) corresponding to 50 mg/dL in whole blood, or less after 48 h of age [1]. Neonatal hypoglycemia is generally defined as a blood glucose value less than 40 mg/dL (2.2 mmol/L) [2]. Hyperinsulinism can be suspected when the plasma insulin concentration is inappropriately normal or elevated for the level of hypoglycemia, and plasma urine ketones as well as free fatty acids are low. In addition, this condition should also be suspected when there is a glycemic response to glucagon at the time of hypoglycemia [3]. Neonatal hyperinsulinemic hypoglycemia (HH) is a leading cause of neonatal care admission. It is characterized by dysregulated insulin secretion and is classified into three main types: transient forms related to perinatal stress, infections, drugs, diffuse or focal nesidioblastosis, monogenic forms due to single-gene defects involved in insulin secretion, and those associated with syndromes such as overgrowth syndromes, like Beckwith-Wiedemann syndrome, or post-natal growth failure syndromes like RASopathies, and Kabuki make-up syndrome (KS) [4]. The exact and sometimes overlapping molecular mechanisms leading to hypoglycemia in these syndromes have not fully been elucidated, although hyperinsulinism, augmented energy consumption, and dysregulation of growth hormone and cortisol have been reported [5, 6]. Early intervention is essential to minimize the risk of poor neurologic outcomes and developmental delay [7, 8]. Moreover, since peculiar dysmorphic signs might be mild at birth and in early childhood, both neonatologists and pediatricians should train to look for and recognize them [9, 10]. Since the first patients with KS reported at our Department 30 years ago [11], we have encountered other patients [12], and recently a KS presenting with facial dysmorphisms, neonatal hypotonia, cerebral anomalies, feeding difficulties, and neonatal HH responsive to Diazoxide. Very recently, diagnostic criteria for KS, by an international consensus, have been established [13]. A definite diagnosis of KS can be made in a male or female patient of any age with a history of infantile hypotonia, developmental delay and/or intellectual disability, and one or both of the following major criteria: 1) A pathogenic or likely pathogenic variant in Lysine (K)-specific methyl transferase 2D (*KMT2D*, MIM# 602113) on chromosome 12q13, linked to Kabuki syndrome subtype 1 (KS1, MIM# 147920), or in lysine(K)-specific demethylase 6A (*KDM6A*, MIM# 300128) on chromosome Xp11, linked to Kabuki syndrome subtype 2 (KS2, MIM# 300867), 2) Typical dysmorphic features (resembling the peculiar make-up mask) including long palpebral fissures (a palpebral fissure measurement greater than or equal to 2 SD above the mean for age) with eversion of the lateral third of the lower eyelid and two or more of the following: arched and broad eyebrows with the lateral third displaying notching or sparseness; short columella with depressed nasal tip; large, prominent or cupped ears; persistent fingertip pads. Less frequent findings include skeletal anomalies (deformed spinal column with or without sagittal cleft vertebrae and brachydactyly), dermatoglyphic abnormalities, mild to moderate mental retardation, postnatal growth deficiency, visceral abnormalities, premature thelarche in girls, and susceptibility to infections due to immunodeficiency.

## Patient presentation

Since our patient was born preterm at 35 completed weeks gestation, in this report we have referred to both chronological age (CrA), and corrected age for prematurity (CA) considering the difference of 35 days to reach the 280 days length of full-term pregnancy.

**Fig. 1:**
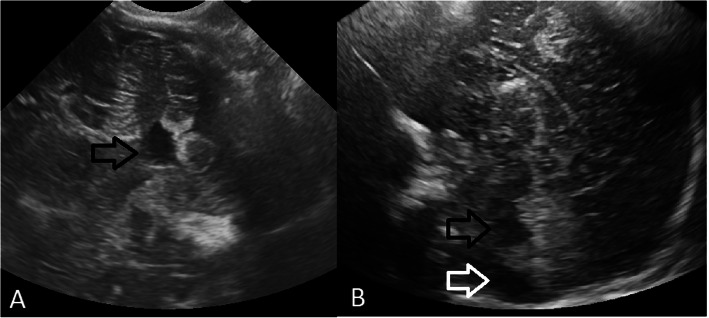
Brain ultrasound. **a**. Axial view through the mastoid fontanel: enlarged fourth ventricle (black arrow). **b**. Sagittal view through the anterior fontanel: cerebellar vermis hypoplasia with secondary enlarged of the fourth ventricle (black arrow) and enlarged *cisterna magna* (white arrow)

**Fig. 2:**
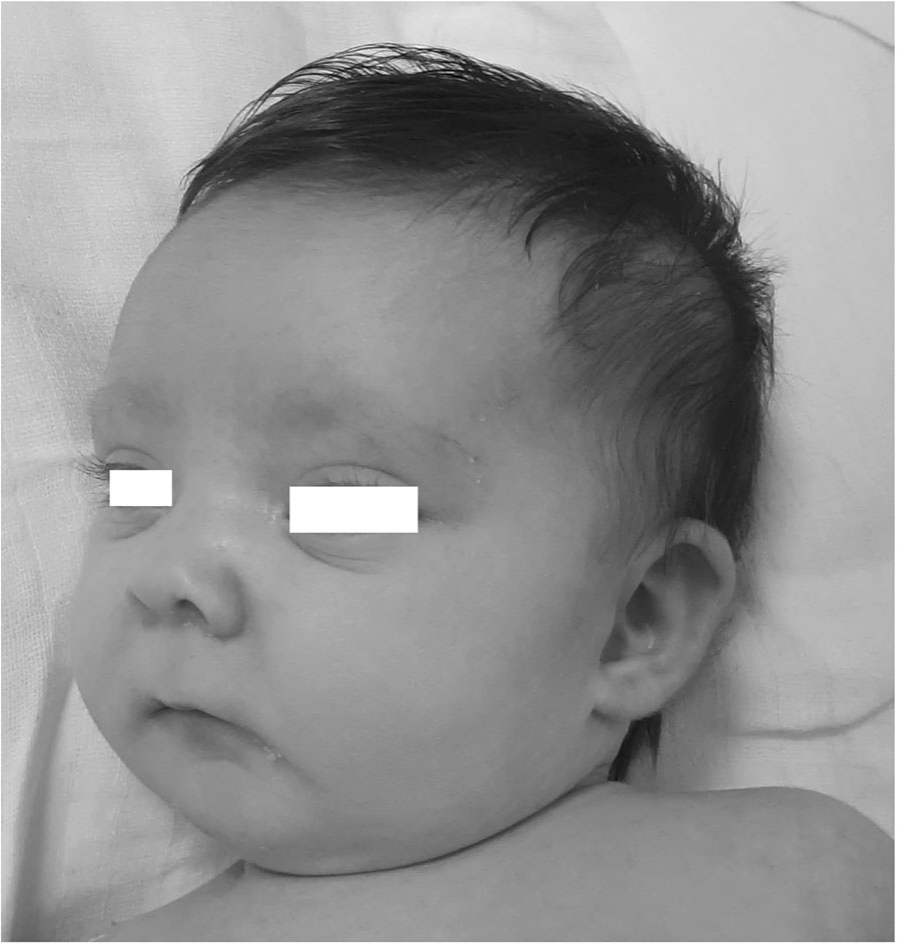
Dysmorphic facial features suggestive of Kabuki syndrome

**Fig. 3:**
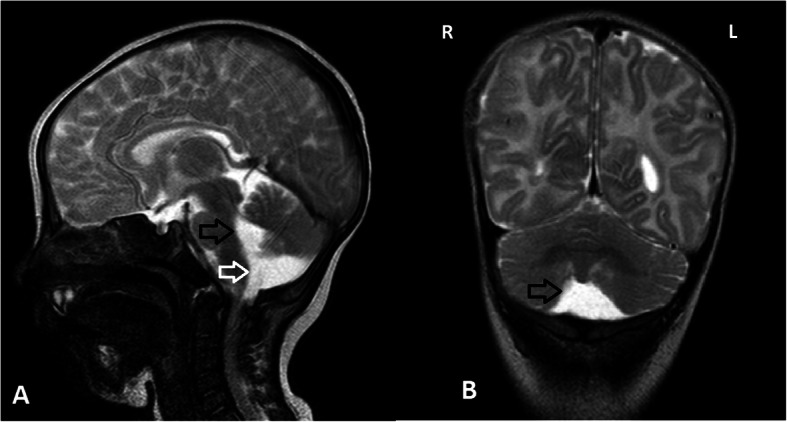
T2 weighted FSE Brain Magnetic Resonance Imaging. **a**. Sagittal scan: enlarged fourth ventricle (black arrow), and *cisterna magna* with inferior vermian hypoplasia (white arrow). **b**. Coronal scan: Enlarged *vallecula cerebelli* and hypoplasia of the cerebellar hemispheres (black arrow)

Our patient is an Italian female neonate, the first child of nonconsanguineous healthy parents, born at 35 weeks of gestation by elective cesarean section for preterm premature rupture of membranes and breech presentation. Except for an underweighting mother (BMI 16.5), the pregnancy was otherwise uneventful. The Apgar scores were 8 and 9 at 1 and 5 min, respectively. Her birth weight was 2755 g (89th centile), length 47 cm (84th centile) and occipitofrontal circumference (OFC) 33 cm (84th centile). She was transferred at 12 h of life for prematurity and persistent hypoglycemia not responsive to enteral feeding to our Neonatal Intensive Care Unit (UTIN). On physical examination, she showed mild dysmorphic facial features that became highly suggestive of a syndromic condition at around 15 days of life: long palpebral fissures, arched eyebrows with sparse outer lateral half, anteverted nares, short columella with depressed nasal tip, and thin vermillion of the upper lip. Other findings were high-arched palate, retrognathia, short neck, brachydactyly, joint hypermobility, right hand single palmar crease, and sacral dimple. At admission neurologic examination revealed generalized hypotonia of central origin, weak cry, reduced reactivity with impairment of sucking and swallowing. Thus, a nasogastric tube was inserted for feeding. Severe hypoglycemia was confirmed (29 mg/dL; equivalent to 1.6 mmol/L), and 200 mg/kg intravenous bolus of 10% dextrose, followed by continuous infusion (8 mg/kg/min) was given. Persistent hypoglycemia (< 40 mg/dL) was not responsive to intravenous 10% dextrose infusion up to 20 mg/kg/min and concomitant milk feeding, providing an adequate caloric intake. Plasmatic adrenocorticotropic hormone (ACTH), cortisol, basal insulin, C peptide, Growth hormone (GH) were normal, urine ketones were absent and free fatty acids were low. Thus, on day 15, a glucagon stimulation test was performed. After basal glycemia (32 mg/dL) was measured (T0), intramuscular Glucagon (0.5 mg IM) was administered causing an increase in glycemic values at T15 (75 mg/dL), and T30 (89 mg/dL), thus, confirming the clinical suspicion of hyperinsulinism. Then, treatment with oral diazoxide was started with 7 mg/kg divided in 3 daily doses, and on day 19, increased to 10 mg/kg/day. After 6 doses glycemic values over the suggested cut-off point (63 mg/dL), were finally achieved [1]. Neonatal screening revealed a congenital hypothyroidism, confirmed on day 5 (TSH 28.7 mIU/L, fT4 0.86 ng/L, fT3 2.39 ng/L), treated with levothyroxine 10 μg/kg/day. Thyroid US was normal. Transitory hypocalcemia, with normal parathyroid hormone values (26.1 ng/L), was responsive to slow bolus infusion of 10% calcium gluconate and subsequent oral therapy lasting one week. Cardiac ultrasound assessment revealed an interventricular septum defect, restrictive during hospital stay, and accessory *chordae tendineae* without hemodynamic alterations (neither mitral regurgitation, nor left ventricle outflow obstruction). Ophthalmological examination and evoked otoacoustic emissions screening were normal. Brain ultrasonography (US) performed on day 15, showed hypoplasia of the cerebellar vermis with enlarged fourth ventricle and *cisterna magna* (Fig. 1). Since facial dysmorphic features (Fig. 2) were highly suggestive of Kabuki syndrome, whole-exome sequence analysis was carried out in the proband and her parents. In the patient a novel heterozygous acceptor splicing-site mutation c.674-1G > A in *KMT2D* gene was identified. The pathogenetic variant of disease associated gene in the patient was confirmed by Sanger sequencing. The father was carrying a heterozygous mutation c.1441C > T (p.Arg481Ter) in pantothenate kinase 2 (*PANK2*) (Hallervorden-Spatz syndrome), transmitted to the daughter. Sanger sequencing did not reveal alterations of *PANK2* gene in the mother. Since the latter is a recessive disorder and not related to the clinical profile, no further genetic investigation was considered in the patient. On request of the mother, in relation to family logistical difficulties of managing the child at home due to lockdown restriction for COVID-19, they remained in our hospice until the child was 3 months CrA (1 months and 25 days CA). A brain MR at 1 months and 24 days CA confirmed the hypoplasia of cerebellar vermis (Fig. 3). At the last follow up evaluation at 4.5 months CrA (3 months and 10 days CA) she was bottle fed with formula milk, and maintained adequate glycemic values with 8 mg/kg/day of diazoxide. Levothyroxine treatment has been effective to normalize plasmatic TSH and fT4 values. Nevertheless, she showed a postnatal growth failure involving weight 4270 g (− 2.78 SD), and OFC 37 cm (− 2.33 SD), with relative sparing of length equal to 60 cm (38th centile; − 0.3 SD). In front of a further reduction of length centile, IGF-1 and GH assessments have been scheduled. Her global development is slightly delayed, with persistent mild generalized hypotonia causing in prone position inability to extend in the thorax area, delayed achievement of social smile (3 months CA) and absence of reciprocal vocalization. A home monitoring program of glycemic values, diazoxide dosage and alimentary regimen has been started with her parents and the reference pediatrician, obtaining adequate glycemic control. She has been enrolled in a neurodevelopmental multidisciplinary follow-up program.

## Discussion and conclusions

Our patient presented with a classical KS neonatal phenotype, consisting in facial dysmorphisms, congenital hypotonia of central type and feeding difficulties. Cerebellar vermis hypoplasia early identified by US, has been described as an occasional finding in KS [14]. HH constituted the main neonatal clinical challenge and was only responsive to diazoxide treatment. KS with an incidence about 1/32000 [15], is caused by *KMT2D* (KS1), and *KDM6A* (KS2) pathogenic variants in 70% and in 5% of patients, respectively [16, 17]. More than 600 *KMT2D* mutations in the whole gene have been recently identified, including nonsense, indels, splicing-sites, frameshift and missense variants leading to truncated proteins [18]. A small number of Ras-related protein 1A (*RAP1A*, MIM# 179520), Ras-related protein 1B (*RAP1B,* MIM# 179530) and Heterogeneous Nuclear Ribonucleoprotein K (*HNRNPK*, MIM# 600712), mutations has recently been reported to be associated with a condition partially overlapping or suggestive of Kabuki syndrome [19–21]. KMT2D and KDM6A are large, enzymatically active scaffold proteins (histone methyltransferases and chromatin-bound protein), that form the core of nuclear regulatory structures of COMPASS complex (complex of protein associated with Set-1) like family, that enhance gene expression of specific loci via the targeted modification of histone-3 tail residues, promoting active euchromatic conformations and interacting with other receptors (transcription promoting enhanceosomes). Other key COMPASS complex genes than *KMT2D* and *KDM6A*, have been linked to human congenital syndromes with postnatal growth restriction as Rubinstein-Taybi type 1 (*CBP*) and type 2 (*EP300*) and Kleefstra syndrome type 2 (*KMT2C*), whereas other DNA methylation defects have been described up to 100% of several mono/oligogenic diseases responsible for constitutional neurodevelopmental disorders as Fragile X syndrome, Sotos syndrome, Tatton-Brown-Rahman syndrome and Kagami-Ogata syndrome [22, 23]. Furthermore, a homologue of *KDM6A* called *KDM6C* (*UTY*; MIM# 400009), another H3K27 demethylase, is located on the Y-chromosome [24] and constitutes a possible candidate gene for KS in male individuals [18]. Aberrations of the mitogen-activated protein kinase (MAPK) signaling pathway in zebrafish morphants for kmt2d and rap1, as well as Kmt2d knock out mices have also been reported [20]. A lower incidence of hypoglycemia in *KMT2D* compared to *KDM6A* variants, respectively 3.5 and 21.8% has been recently reported [25]. In our patient persistent hypoglycemia represented the main neonatal emergency. Any newborn presenting with persistent hypoglycemia should have urgent investigations to establish the cause and key step in the assessment involves determining the intravenous glucose infusion rate required to maintain normoglycemia. A glucose infusion rate of more than 8 mg/kg/min is highly suggestive of HH. In our patient persistent low glycemic values (< 40 mg/dl; equivalent to 2.2 mmol/L) despite high dextrose infusion up to 20 mg/kg/min, and the rapid response to diazoxide confirmed HH. Up to 6% of KS neonates present with HH [4]. Since HH in patients with KS is well managed medically, a timely recognition of hyperinsulinemic episodes will improve outcomes, and prevent aggravation of the preexisting mild to moderate intellectual disability [6]. Diazoxide, the first-line pharmacologic treatment is, a potassium channel agonist that binds to the sulfonylurea receptor component of the beta cell’s KATP channel, resulting in hyperpolarization of the plasma membrane and cessation of insulin secretion [26]. No differences were observed in the responsiveness to diazoxide effect between *KMT2D* and *KDM6A* variants [25]. It is administered orally with gradual dose titration up to 10–15 mg/kg/day divided 3 times daily [27]. A gradually increasing dose approach was preferred in our preterm patient in light of the high risk of ductus arteriosus dilatation and necrotizing enterocolitis in preterm neonates that could have a contributory effect (like perinatal stress, or intestinal malformation) [28, 29]. A standard length of diazoxide treatment has not been well established, and often appears to resolve during the first decade of life, since the individual response is dependent of the interaction of several conditions [30]. Cerebellar vermis hypoplasia and nodular heterotopia could be related to functional inhibition of neural crest development by *KMT2D* loss-of-function as recently reported suggesting that KS could be a neurocristopathy [31]. Moreover, KMT2D deficiency disrupts neurogenesis by negatively affecting neural stem/progenitor cells (NSPC) maintenance functions, including cell cycle, proliferation, and survival, accompanied by decreased adult NSPC numbers and precocious neuronal differentiation [32]. Neurodevelopmental profile in our patient with KS1 was characterized by a progressive reduction of OFC and cerebellar vermis hypoplasia concomitant to developmental delay and generalized hypotonia with oromotor dysfunction. Postnatal microcephaly has been reported in 32% of patients with KS1 [33]. Developmental delay and/or intellectual disability have been considered diagnostic criteria in a recent consensus [13]. Generalized hypotonia has been described in 100% of subjects with cerebellar vermis hypoplasia [34]. In our patient an interventricular septum defect and accessory *chordae tendineae* without hemodynamic alterations were the only left ventricle anomalies. In patients with KS1 in comparison to KS2 a higher frequency of heart defects, around 70% versus 45%, with prevalent left ventricle involvement have been reported [35]. Although at birth our patient, showed a normal intrauterine growth pattern, we have observed a progressive postnatal growth failure, involving weight and OFC, with values < 2 SD. This growth pattern has been previously reported in KS [36]. Moreover, the KS1 growth impairment has been recently associated with a “decelerated epigenetic aging” profile secondary to disrupting mutations in epigenetic regulatory molecules [37]. A recent study identified a GH deficiency in 13% of subjects with KS1, with a size reduction beyond the predicted one (− 2 SD and − 1.8 SD for males and females, respectively). Interestingly in this study an absent response to GH therapy has been documented [33]. Growth during childhood depends primarily on the GH/IGF-1 axis and thyroid hormones. Since in our patient a primary hypothyroidism has been early identified and treated, with TSH and fT4 values normalization, in front of a further reduction of length centile and to monitor the described potential abnormalities in hypothalamic pituitary axis [38], IGF-1 and GH assessments could be considered. Since growth impairment has been widely described a strict auxological monitoring should be ensured mainly in the first three years of life and growth charts for KS1 should be adopted [33]. Hearing loss, conductive, or mixed mainly due to recurrent otitis media, should be ruled out since they have been reported in KS with a frequency up to 76.9% [39]. Different cancer types have been associated with KS, in childhood too [40] and likely driven by the same hyperactivation of RAS/MAPK signaling responsible for the development of both benign neurofibromas and malignant plexiform neurofibromas described in Neurofibromatosis type 1 [41, 42]. Recently new treatments based on knowledge of epigenetic pathomechanisms, as those related to small molecule inhibition of RAS/MAPK signaling, have been proposed [43]. In our patient a home monitoring program of glycemic values, diazoxide dosage and alimentary regimen has been started with her parents and the reference pediatrician, obtaining adequate glycemic control. She has been enrolled in a neurodevelopmental multidisciplinary follow-up program.

Neonatologists and pediatricians should enhance their ability to recognize clinical dysmorphic features and complex phenotypes suggestive of genetic conditions by a specific training in clinical genetics. Adoption of the so called “diagnostic handles”, a wider clinical competence in pediatric neurological and developmental assessment, brain imaging and neurophysiological findings could allow an early diagnosis, aiming to ensure a rapid enrollment in a multidisciplinary and individualized follow-up for prevention and early intervention in the several clinical domains potentially involved.

## Data Availability

The clinical data used during the current study are available from the corresponding author on reasonable request.

## Abbreviations

ACTH: Adrenocorticotropic hormone
BMI: Body mass index
CA: Corrected age
COMPASS complex: Complex of protein associated with Set-1
CrA: Chronological age
GH: Growth hormone
HH: Hyperinsulinemic hypoglycemia
*HNRNPK*: Heterogeneous Nuclear Ribonucleoprotein K
IGF-1: Insulin growth factor 1
KATP: ATP-sensitive potassium channel
*KDM6A*: Lysine(K)-specific demethylase 6A
*KMT2D*: Lysine (K)-specific methyl transferase 2D
KS: Kabuki syndrome
MAPK: Mitogen-activated protein kinase
NSPC: Neural stem/progenitor cells
OFC: Occipitofrontal circumference
*RAP1A*: Ras-related protein 1A
*RAP1B*: Ras-related protein 1B
US: Ultrasonography
